# Epidemiological analysis of the first 1000 cases of SARS‐CoV‐2 lineage BA.1 (B.1.1.529, Omicron) compared with co‐circulating Delta in Wales, UK

**DOI:** 10.1111/irv.13021

**Published:** 2022-07-12

**Authors:** Nicole Pacchiarini, Clare Sawyer, Christopher Williams, Daryn Sutton, Christopher Roberts, Felicity Simkin, Grace King, Victoria McClure, Simon Cottrell, Helen Clayton, Andrew Beazer, Catie Williams, Sara M. Rey, Thomas R. Connor, Catherine Moore

**Affiliations:** ^1^ Communicable Disease Surveillance Centre (CDSC) Public Health Wales Cardiff Wales UK; ^2^ Informatics Division, Operations and Finance Directorate Public Health Wales Cardiff Wales UK; ^3^ Pathogen Genomics Unit Public Health Wales Cardiff Wales UK; ^4^ Cardiff University School of Biosciences Cardiff University Cardiff Wales UK; ^5^ Wales Specialist Virology Centre, Microbiology Public Health Wales Cardiff Wales UK

**Keywords:** COVID‐19, Genomics, Omicron, SARS‐CoV‐2, Surveillance, Wales

## Abstract

**Background:**

The Omicron (lineage B.1.1.529) variant of severe acute respiratory syndrome coronavirus 2 (SARS‐CoV‐2) was first reported in Wales, UK, on 3 December 2021. The aim of the study was to describe the first 1000 cases of the Omicron variant by demographic, vaccination status, travel and severe outcome status and compare this to contemporaneous cases of the Delta variant.

**Methods:**

Testing, typing and contact tracing data were collected by Public Health Wales and analysis undertaken by the Communicable Disease Surveillance Centre (CDSC). Risk ratios for demographic factors and symptoms were calculated comparing Omicron cases to Delta cases identified over the same time period.

**Results:**

By 14 December 2021, 1000 cases of the Omicron variant had been identified in Wales. Of the first 1000, just 3% of cases had a prior history of travel revealing rapid community transmission. A higher proportion of Omicron cases were identified in individuals aged 20–39, and most cases were double vaccinated (65.9%) or boosted (15.7%). Age‐adjusted analysis also revealed that Omicron cases were less likely to be hospitalised (0.4%) or report symptoms (60.8%). Specifically a significant reduction was observed in the proportion of Omicron cases reporting anosmia (8.9%).

**Conclusion:**

Key findings include a lower risk of anosmia and a reduced risk of hospitalisation in the first 1000 Omicron cases compared with co‐circulating Delta cases. We also identify that existing measures for travel restrictions to control importations of new variants identified outside the United Kingdom did not prevent the rapid ingress of Omicron within Wales.

## 1 INTRODUCTION

On the 23rd of November,^1^ a new lineage of SARS‐CoV‐2 with an unusual number of mutations was identified from four publicly released genomes, all of which had links to South Africa. This lineage, B.1.1.529, was subsequently identified in samples collected on 9 November 2021^2^ in South Africa, following a global alert that was issued on November 25. Subsequently, the lineage was rapidly assigned Variant of Concern (VOC) status by both the World Health Organisation (WHO) and national public health agencies, due to a combination of its potential for immune escape, and a likely transmission advantage, as evidenced by a rapid increase in cases numbers seen in South Africa, associated with this lineage.

Following the identification of Omicron, in Wales, reflex PCR testing and targeted whole genome sequencing (WGS) were put in place to allow screening of positives with a known travel link to South Africa and other countries where Omicron had been reported. Since the start of the pandemic, Wales has operated a WGS‐based surveillance system, which has seen ~23% of all positive Welsh SARS‐CoV‐2 cases sequenced. The first case of the Omicron variant in Wales was reported on 3 December 2021 from a sample collected from an unvaccinated traveller arriving from South Africa. Initial identification of Omicron was performed by the Wales Specialist Virology Centre (WSVC), Public Health Wales (PHW) Microbiology Cardiff, through a routinely used reflex variant PCR assay. The result was confirmed using WGS by the PHW Pathogen Genomics Unit (PenGU). Onward transmission from this case was confirmed in six contacts (all six genotypically confirmed, four sequenced). Whereas the first reported case through the priority testing route was identified at the start of December, routine WGS subsequently identified a confirmed case in Wales who had a specimen collected on November 29. This individual was double vaccinated, aged 30–39, and had no international travel or links to international travel, and so would not have been captured by a case definition that included travel as a component. Two close contacts were subsequently also confirmed.

By 14 December 2021, over 1000 genotypically and/or whole genome sequenced confirmed cases had been detected in Wales. We summarise these first 1000 confirmed cases by underlying test results, demographics, vaccination status, travel and severe outcome status [hospitalisation/admission to intensive care unit (ICU)/death]. 

## 2 METHOD

### 2.1 Surveillance of COVID‐19 variants of concern/variants under investigation in Wales: Methods

In Wales, PCR tests for COVID‐19 are free for those with symptoms. An individual can book a PCR test through the UK government portal with any of a new continuous cough, a high temperature or a loss of/change to the ability to smell/taste. Individuals regardless of symptom status can also have a PCR test at a walk‐in testing site. Whereas a proportion of these community samples are tested within the NHS laboratory network in Wales, the majority are tested in the UK Health Security Agency (UKHSA) lighthouse laboratory network. The referral pathway for hospitalised patients differs to the community pathway, with all SARS‐CoV‐2 testing occurring within the NHS Wales laboratory network.

Until 7 January 2022, in Wales, individuals arriving in the United Kingdom from overseas were required to undertake a PCR test 2 and 8 days after their arrival. As well as PCR testing, antigen tests [lateral flow tests (LFTs)] are freely available in the United Kingdom via pharmacies or the UK Government portal. Prior to 7 January 2022, all individuals with a positive antigen LFT were encouraged to have a confirmatory PCR test, regardless of symptom status.

In Wales, Variants of Concern (VOCs) and Variants under Investigation (VUIs) of SARS‐CoV‐2 are identified by either genotyping or WGS. All positive samples within the NHS Wales laboratory network at this time, in response to Omicron, were subjected to reflex PCR testing for key spike mutations associated with VOC/VUIs using the Allplex SARS‐CoV‐2 Variant PCR assays (Seegene, Seoul, Korea) and subsequently sent for confirmatory WGS.^3^ Positive samples from residents of Wales were mostly tested in the UKHSA lighthouse laboratory based at Newport Imperial Park 5 (IP5) in Wales. For positive samples originating from both Welsh NHS labs and from IP5, diagnostic residuals or second samples were passed to the PenGU for sequencing and analysis. A small proportion of Welsh tests were also sent to other parts of the lighthouse lab network, where, depending on site, they could be tested at that laboratory by either a reflex variant PCR or would be passed on for WGS at the Wellcome Sanger Institute, and results reported back to the Welsh NHS system. The cumulative WGS coverage of Welsh SARS‐CoV‐2 cases up to the 20th of December over the course of the pandemic was 24.7%, and over the course of the 3 weeks from the 29th of November to the 19th of December, 29.4% of all Welsh cases were sequenced, with VUI/VOC cases being escalated in real time to surveillance and health protection colleagues for onward action.

Within Wales, the Test, Trace and Protect (TTP) service undertakes contact tracing for all PCR positive COVID‐19 cases. From the initial case, until 22 December 2021, enhanced contact tracing and testing of contacts were undertaken for cases of Omicron. Cases were asked about their symptoms, exposures and contacts, and information was entered in to TTP's contact tracing system.

Vaccination status of confirmed cases was ascertained using the COVID‐19 vaccination register in Wales (the Welsh Immunisation System), which contains all NHS‐registered individuals resident in Wales and vaccination details. An individual was considered to be ‘vaccinated’ (with two doses) if they had had two doses of vaccine 14 days prior to their sample date.

Case admission to hospital status was based on data from ICNET, a hospital infection control data system used across Wales for case management by infection prevention and control (IPC) teams and for systematic surveillance by PHW. An admission was classified as an individual with a positive PCR result for COVID‐19, sequenced as Omicron, who was admitted to hospital on or 1 day before the day of their first positive test or in the 28 days following a positive test. Likewise, admission to ICU status was based on an individual being identified in ICNET as having been admitted to ITU on or 1 day before the day of their first positive test or in the 28 days following a positive test.

Vital status was determined by linking to the Public Health Wales' Rapid Mortality Surveillance data. This surveillance is based on clinician reported deaths in confirmed cases of COVID‐19 from hospitals or care homes, where the clinician suspects COVID‐19 is a causative or contributory factor in the death. We carried out confirmation of deaths against ICD10‐coded death certificates from the Office of National Statistics (ONS) in order to identify additional deaths, which may have occurred outside these settings.

Descriptive analysis was undertaken in R Studio, and risk ratios (hospitalisation and symptom status adjusted for age), confidence intervals and *P*‐values were calculated in STATA 14.

## 3 RESULTS

### 3.1 Descriptive epidemiology of Omicron cases in Wales, 29 November 2021 to 14 December 2021

**FIGURE 1:**
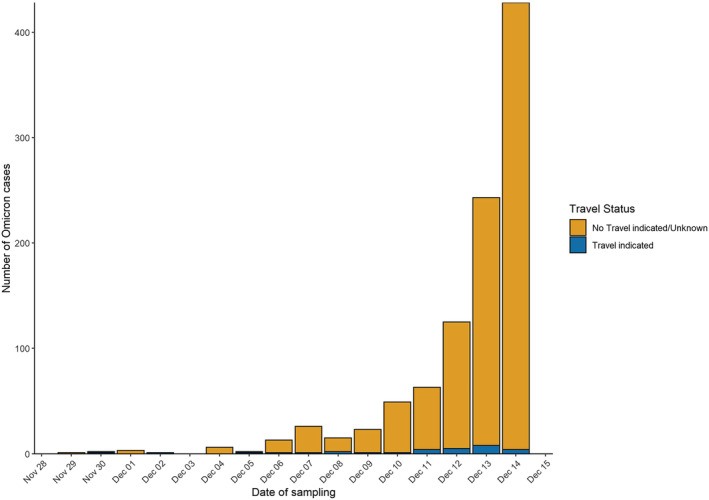
Sample date for SARS‐CoV‐2 Omicron cases by travel status of resident, Wales, 29 November to 14 December 2021, data as of 30 December 2021 (*n* = 1000)

Of the 1000 cases of confirmed Omicron analysed up to the 14 December (Figure 1), 251 (25%) were detected via WGS by PHW's PenGU, nine by WGS from private laboratories outside Wales (0.9%) and 206 (20.6%) by genotyping alone, and over half (534, 53.4%) were both genotyped and sequenced.

**FIGURE 2:**
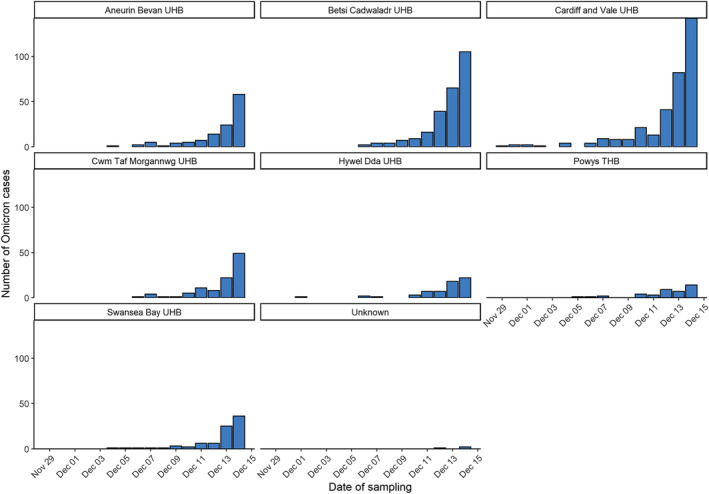
Sample date for SARS‐CoV‐2 Omicron cases by local health board of resident, Wales, 29 November to 14 December 2021, data as of 30 December 2021 (*n* = 1000)

Cases were first identified in residents of Cardiff and Vale University Health Board (UHB) (Figure 2). Overall, the age of cases ranged from <1 to 86 years (median: 31 years), and approximately half (52.5%) were female (Figure 2). Overall, 3% (30/970) of individuals reported overseas travel. The most frequently reported destinations were the United Arab Emirates (*n* = 12), Sri Lanka (*n* = 7), South Africa (*n* = 2) and Germany (*n* = 2).

Vaccination status was available for 90% of individuals (*n* = 902). Over two‐thirds of Omicron cases were fully vaccinated (*n* = 659), 36 cases had received only one dose of vaccine, and 161 cases had received a booster. Around 5% of cases were unvaccinated (*n* = 46).

Contact tracing data indicated that 13% (*n* = 131) of the first 1000 Omicron cases were key workers. The most frequently reported key worker groups were ‘Health and Social Care’ (*n* = 47), followed by ‘Key Public Service’ (*n* = 23) and Education and Childcare (*n* = 22).

### 3.2 Epidemiology of Omicron and Delta cases in Wales, 29 November 2021 to 14 December 2021

**FIGURE 3:**
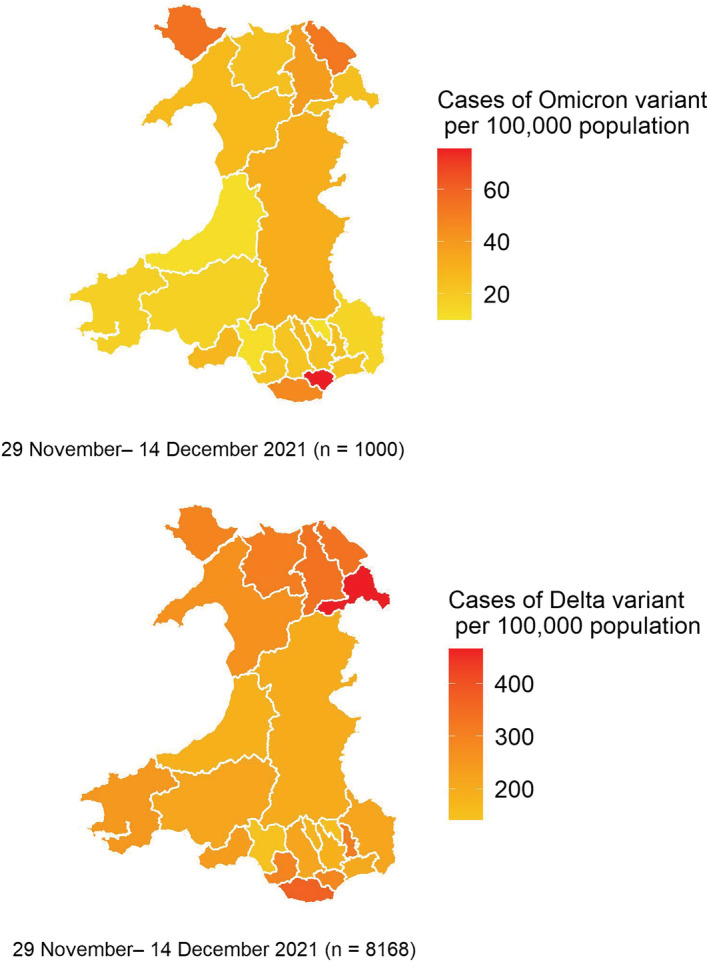
Geographical distribution of Omicron and Delta variant cases per 100 000 population, Wales, 29 November to 14 December 2021, data as of 30 December 2021

There were 8168 Delta cases identified within the same period as the first 1000 Omicron cases. The first cases of confirmed Omicron were identified in the local authority of Cardiff, and by December 14, Cardiff had a prevalence of over 60 Omicron cases per 100 000 of the population (Figure 3). In contrast, at this time, the highest rates per 100 000 of the Delta variant (including VUI‐21OCT‐01, AY.4.2) were seen in Wrexham in North Wales, where rates were over 400 Delta cases per 100 000 of the population (Figure 3).

Enhanced investigations by multidisciplinary incident management teams identified that Omicron cases in South Wales reported having contacts who lived in cities in England, such as London and Birmingham. This is in line with early modelling work that estimated London to have the highest weighted prevalence as well as the first cases of Omicron.^4^, ^5^

Reported clinical symptoms differed between cases with confirmed Omicron and those reported by cases with confirmed Delta (Table 1). Four individuals with Omicron (0.4%) were admitted to hospital within 28 days of their positive test, all had a mild course with no requirement for intensive care, and all were discharged home within 28 days. In contrast, 107 patients with Delta were admitted to hospital (1.3%), with 14 (0.2%) admitted to intensive care. Thirty‐two people with Delta died within 28 days of their sample collection date, 87.5% (28/32) of whom had COVID‐19/respiratory infection listed amongst ICD10‐coded causes of death.

**TABLE 1:** Characteristics of SARS‐CoV‐2 Delta variant cases collected within the same time period as the first 1000 Omicron variant cases, Wales, 29 November to 14 December 2021, data as of 30 December 2021

	Number and percentage of Delta variant cases^a^ (*n* = 8168)		Number and percentage of Omicron variant cases^b^ (*n* = 1000)		Risk ratio (RR)	95% CI	*P*‐value
	*n*	%	*n*	%			
Age group (years)							
0–9	1089	13.3%	16	1.6%	0.09	0.06–0.15	*P* < 0.05
10–19	1513	18.5%	100	10.0%	0.7	0.5–0.8	*P* < 0.05
20–29	839	10.3%	352	35.2%	3.6	3.2–4.1	*P* < 0.05
30–39	1417	17.3%	224	22.4%	1.3	1.2–1.5	*P* < 0.05
40–49	1401	17.2%	144	14.4%	0.8	0.7–1.0	*P* < 0.05
50–59	1221	14.9%	108	10.8%	0.7	0.6–0.9	*P* < 0.05
60–69	485	5.9%	45	4.5%	0.8	0.6–1.0	0.07
70+	203	2.5%	11	1.1%	0.5	0.3–0.8	*P* < 0.05
Sex							
Male	3976	48.7%	462	46.2%	1.1	1.0–1.2	*P* = 0.2
Female	4150	50.8%	525	52.5%			
Unknown	42	0.5%	13	1.3%	NA	NA	NA
Travel history							
Yes	70	0.90%	30	3.0%	2.8	2.1–3.8	*P* < 0.05
No travel indicated/unknown	8098	99.1%	970	97.0%			
Vaccination status							
Unvaccinated	1356	16.6%	46	4.6%	0.3	0.2–0.4	*P* < 0.05
1 dose	572	7.0%	36	3.6%	0.5	0.4–0.7	*P* < 0.05
2 dose	3947	48.3%	659	65.9%	2.0	1.7–2.2	*P* < 0.05
Booster	671	8.2%	157	15.7%	1.9	1.6–2.2	*P* < 0.05
Unknown	1622	19.9%	102	10.2%	NA	NA	NA
Symptomatic (self‐reported)^c^							
Yes	5403	66.1%	608	60.8%	0.9	0.8–1.0	*P* < 0.05
No	2405	29.4%	310	31.0%			
Unknown	360	4.4%	82	8.2%	NA	NA	NA
Symptoms (self‐reported)^c^							
Fever	2280	27.9%	315	31.5%	1.2	1.1–1.4	*P* < 0.05
Cough	3699	45.3%	461	46.1%	1.1	1.0–1.2	*P* = 0.29
Runny nose	1548	19.0%	155	15.5%	0.8	0.7–1.0	*P* < 0.05
Sneezing	1110	13.6%	124	12.4%	0.8	0.7–1.0	*P* < 0.05
Anosmia	2106	25.8%	89	8.9%	0.3	0.2–0.3	*P* < 0.05
Hospitalisation (admitted within 28 days of sample date)^c^							
Yes	107	1.3%	4	0.4%	0.3	0.1–0.9	*P* < 0.05
No	8061	98.7%	996	99.6%			
Intensive care unit							
Yes	14	0.2%	0	0.0%	‐	‐	‐
Death							
Yes	32	0.4%	0	0.0%	‐	‐	‐

^a^ Confirmed using WGS only.

^b^ Confirmed using both genotyping and WGS techniques.

^c^ For hospitalisation, symptomatic status and symptoms, the RRs were adjusted for age group.

## 4 DISCUSSION

Following the identification and confirmation of the first case infected with the Omicron variant of SARS CoV‐2 in Wales on 3 December 2021, there was a rapid increase in the number of confirmed cases with evidence of unidentified community transmission. As of 29 December 2021, Omicron was confirmed as the dominant variant of SARS‐CoV‐2 circulating in Wales.^6^ As seen in other European nations, whereas initial cases were identified in the capital, Cardiff, within a short time frame, cases were spread across all areas of Wales.^7^

All of the first 1000 cases of Omicron in Wales were classified as the BA.1 lineage, based on WGS or based on the presence of the 69/70 deletion (which leads to the S gene target failure)—a characteristic of BA.1, used to identify it.^8^ The first sequenced case of BA.2 in Wales was identified in a sample taken on 30 December 2021, after the first 1000 BA.1 cases had been detected.

We have identified that a higher proportion of Omicron cases were aged 20–29 years old (RR: 3.6, 95% CI: 3.2–4.1, *P* < 0.05) and 30–39 years old (RR 1.3 95% CI: 1.2–1.5, *P* < 0.05) and no difference by sex (RR: 1.1, 95% CI: 1.0–1.2, *P* = 0.2) compared with Delta cases during the same time frame. Furthermore, compared with Delta cases identified during the same time frame, an increased risk ratio was seen in individuals who had been vaccinated with two doses (RR 2.0, 95% CI: 1.7–2.2, *P* < 0.05) or the booster (RR 1.9, 95% CI: 1.6–2.2, *P* < 0.05) in the first 1000 Omicron cases (Table 1). This is in line with recent work identifying that two vaccine doses only offer short‐term protection against symptomatic Omicron infection.^9^, ^10^ It is important to note, however, that this finding is likely to be biased—the majority of the first 1000 cases of Omicron, being individuals aged 20–40 years, all would have been offered the second vaccination dose in Wales by this point in time.

Omicron cases were 2.8 times more likely to report overseas travel than Delta cases identified in the same period (95% CI: 2.1–3.8, *P* < 0.05). This is likely due to the targeted testing in arriving travellers as part of the initial Omicron response. However, though the response to the detection of Omicron in the United Kingdom included travel restrictions on a number of African countries,^11^ the individual with the earliest sample date in Wales had not travelled and had no identified links to international travel. In addition to this, of the first 1000 Omicron cases in Wales who did report overseas travel, individuals most commonly reported travel to the United Arab Emirates, Sri Lanka and Germany—none of which were on the United Kingdom's ‘red list’^11^ at any point in the response. This suggests that Omicron arrived in Wales prior to the 29th of November—highlighting the rapid international spread of the virus and the likelihood of undetected community transmission within Wales. Although some other nations in Europe identified their earliest cases as arriving travellers from South Africa,^12^ a study from Denmark^7^ identified that initial cases who were arriving travellers were arriving from other European countries with Germany and Spain most frequently reported. This highlights the rapid international spread of Omicron and the limitations of border based restrictions and also demonstrates the limitations of case definitions for enhanced testing that include a travel component, for a variant as transmissible as Omicron.^13^

The following analysis, adjusted for age, revealed an overall reduction in the proportion of Omicron cases reporting any of the main three symptoms (cough, fever, anosmia) (RR: 0.9, CI: 0.8–1.0, *P* < 0.05). We also identified a shift in the symptom profile of Omicron cases, which has implications for testing strategies in the United Kingdom. Although a slightly higher proportion of Omicron cases reported a fever (RR: 1.2, CI: 1.1–1.4, *P* < 0.05), no difference was found between Delta and Omicron cases in terms of reported cough (RR: 1.1, CI: 1.0–1.2, *P* = 0.29). Moreover, a significant reduction was observed in the proportion of Omicron cases reporting anosmia (RR: 0.3, 95% CI: 0.2–0.3, *P* < 0.05), runny nose (RR: 0.8, 95% CI: 0.7–1.0, *P* < 0.05) and sneezing (RR: 0.8, 95% CI: 0.7–1.0, *P* < 0.05) compared with co‐circulating Delta cases (Table 1). This reduction in the proportion of cases reporting anosmia was also described in cases experiencing adjusted sense of smell or taste during an Omicron outbreak in Norway.^12^ This evidence for a reduction in the prevalence of symptoms, particularly anosmia, may be a reason for why undetected Omicron may have been circulating in Wales.

As has been observed elsewhere, this analysis revealed that the risk of hospitalisation for the initial 1000 cases of Omicron, compared with Delta, was reduced.^9^, ^14^, ^15^, ^16^ In this age adjusted analysis, Omicron cases were less likely to be hospitalised compared to Delta cases (RR: 0.3, 95% CI: 0.1–0.9, *P* < 0.05). Furthermore, very few cases of Omicron required hospitalisation within 28 days of their sample date, with none requiring admittance to intensive care and no deaths—unlike Delta, where of the 107 cases hospitalised, 0.2% (*n* = 14) required admittance to intensive care over the same time period.

## 5 LIMITATIONS

It is likely that the underlying risks and exposures of Omicron cases were different to cases of Delta in the community occurring at the same time.

In addition to this, due to guidelines at the time, Omicron cases received more intensive contact tracing than Delta cases. It is therefore possible that asymptomatic household cases were detected in this study for Omicron cases, but, due to testing guidelines, would not have been for Delta cases. Similarly, due to the volume of cases of Delta, less data were collected as part of contact tracing for Delta cases, than for Omicron cases, especially in relation to travel information and symptom profiles. As a result, estimates around the prevalence of symptoms and of travel may be underestimated for Delta cases in this analysis.

## 6 CONCLUSION

Despite an enhanced public health response, the spread of the Omicron variant was rapid, with cases seen in all areas of Wales within 14 days, and dominance achieved more rapidly than with the emergence of Delta. The majority of cases were fully vaccinated or had received a booster dose and not linked to international travel. This suggests that widespread community transmission had occurred prior to the first identified case in Wales, despite travel restrictions focusing on Omicron‐reporting countries and enhanced control measures focused on these countries. Omicron cases were less severe than Delta cases at the same point in time, having a lower prevalence of hospitalisations and a change in symptom profile including a lower proportion of individuals self‐reporting anosmia. They were also more common in those aged 20–39.

We recommend that symptom profiles and the hospitalisation proportions continue to be monitored, in particular where new variants have arisen, even as testing policies change across the United Kingdom. Existing measures for travel restrictions to control importations of new variants identified outside the United Kingdom have not prevented the rapid ingress and spread of the Omicron variant. Both the methods for identifying countries at risk and the mechanisms by which importation is controlled should be reviewed.

## CONFLICT OF INTEREST

None.

## ETHICS STATEMENT

The study presented encompasses two elements. The first of these does not require specific ethical approval, as it focuses on public health/surveillance questions that make use of sequence data and other metadata that is already shared with the wider world as part of the activities of the COG‐UK consortium (https://www.cogconsortium.uk/). COG‐UK data are released and are publicly available via the ENA, GISAID and the COG‐UK website. The element of the work that would/could require ethical approval is the specific examination of outcome data. The use of named patient data in the investigation of communicable disease outbreaks and surveillance of notifiable disease is permitted under Public Health Wales' Establishment Order. Data were held and processed under Public Health Wales' information governance arrangements, in compliance with the Data Protection Act, Caldicott Principles and Public Health Wales guidance on the release of small numbers. No data identifying protected characteristics of an individual were released outside Public Health Wales. The use of the genomic dataset for research purposes is also covered as part of the COG‐UK project protocol, which was approved by the Public Health England Research Support and Governance Office (RSGO) following review by the PHE Research Ethics and Governance Group (REGG).

## AUTHOR CONTRIBUTIONS

**Nicole Pacchiarini:** Conceptualization; formal analysis; software; visualization. **Clare Sawyer:** Conceptualization; formal analysis; software; visualization. **Christopher Williams:** Supervision. **Daryn Sutton:** Data curation; software. **Christopher Roberts:** Data curation; software. **Felicity Simkin:** Data curation; software. **Grace King:** Data curation; software. **Victoria McClure:** Data curation; software. **Simon Cottrell:** Conceptualization; data curation; supervision. **Helen Clayton:** Data curation; software. **Andrew Beazer:** Data curation; software. **Catie Williams:** Data curation; software. **Sara M. Rey:** Data curation; software. **Thomas R. Connorz:** Supervision.

## Data Availability

Aggregate data available on request due to privacy/ethical restrictions.
